# DynAMo: A Modular Platform for Monitoring Process, Outcome, and Algorithm-Based Treatment Planning in Psychotherapy

**DOI:** 10.2196/medinform.6808

**Published:** 2017-07-20

**Authors:** Tim Kaiser, Anton Rupert Laireiter

**Affiliations:** ^1^ Psychotherapy Research Group Department of Psychology University of Salzburg Salzburg Austria

**Keywords:** health information management, mental health, mental disorders, psychotherapeutic processes, algorithms

## Abstract

**Background:**

In recent years, the assessment of mental disorders has become more and more personalized. Modern advancements such as Internet-enabled mobile phones and increased computing capacity make it possible to tap sources of information that have long been unavailable to mental health practitioners.

**Objective:**

Software packages that combine algorithm-based treatment planning, process monitoring, and outcome monitoring are scarce. The objective of this study was to assess whether the DynAMo Web application can fill this gap by providing a software solution that can be used by both researchers to conduct state-of-the-art psychotherapy process research and clinicians to plan treatments and monitor psychotherapeutic processes.

**Methods:**

In this paper, we report on the current state of a Web application that can be used for assessing the temporal structure of mental disorders using information on their temporal and synchronous associations. A treatment planning algorithm automatically interprets the data and delivers priority scores of symptoms to practitioners. The application is also capable of monitoring psychotherapeutic processes during therapy and of monitoring treatment outcomes. This application was developed using the R programming language (R Core Team, Vienna) and the Shiny Web application framework (RStudio, Inc, Boston). It is made entirely from open-source software packages and thus is easily extensible.

**Results:**

The capabilities of the proposed application are demonstrated. Case illustrations are provided to exemplify its usefulness in clinical practice.

**Conclusions:**

With the broad availability of Internet-enabled mobile phones and similar devices, collecting data on psychopathology and psychotherapeutic processes has become easier than ever. The proposed application is a valuable tool for capturing, processing, and visualizing these data. The combination of dynamic assessment and process- and outcome monitoring has the potential to improve the efficacy and effectiveness of psychotherapy.

## Introduction

### Background

One of the major strategic objectives of the National Institute of Mental Health is to develop ways to tailor existing and new interventions to optimize outcomes and to foster personalized interventions and strategies for sequencing or combining existing and novel interventions [1]. In practice, this can be accomplished by monitoring individual trajectories of change instead of assuming similar treatment responses for every patient. Promising advances have been made in psychotherapy research toward adhering to this goal, leading to the propagation of scientifically informed clinical practice. In the last 20 years, various process and outcome monitoring systems have been developed [2]. One of these well-established outcome monitoring systems is the Outcome Questionnaire-45.2 (OQ-45.2) [3,4]. Using weekly post-session assessments, this system screens for therapeutic change in the domains of reduced symptom distress, interpersonal functioning, and social role. Additionally, potential for risk factors such as suicidal tendencies, substance abuse, and violence is screened for. The OQ system includes a software application called OQ Analyst. This application makes it possible to administer and evaluate routine outcome questionnaires in psychotherapy; it also enables clinicians to check if the outcomes of a current patient are satisfying, or if a revision of the treatment plan is necessary. If a patient’s outcome is not within the expected range, the software warns the clinician.

The Partners for Change Outcome Management System (PCOMS) [5] also uses outcome ratings that are administered before every session. Furthermore, the quality of the therapeutic alliance is rated after sessions using the Session Rating Scale.

These systems mostly provide mental health practitioners with valuable information on the outcomes of their clinical interventions and on the risk that may worsen a patient's condition. By incorporating this information, practitioners may correct their treatment plan when the patient's condition appears to be “off track.” In addition, patients get motivated by noticing that their improvements are actually measurable. Thus, it is not surprising that systematic monitoring of relevant variables has been found to not only prevent negative treatment outcomes but also to enhance positive ones [6].

A successful psychotherapy continues to show positive outcomes after most of the therapy sessions and also between sessions. Over time, patients begin to form mental representations of their psychotherapy that can be activated between therapy sessions. Following this assumption, Orlinsky et al [7] were the first to propose the “representations of a patient’s psychotherapy between two sessions” as a focus for psychotherapy research, building the foundation of what later became the concept of the intersession process. The intersession process encompasses thoughts, feelings, and behaviors concerning a patient’s current psychotherapy, including the therapist. They occur between two therapy sessions; can be of varying emotional quality, intensity, and frequency; and include memories of words and feelings toward the dyadic partner, applying techniques learned in the psychotherapy or doing therapeutic homework. The intersession process has been operationalized and can be measured for a specific period of time between two sessions using the Intersession Experience Questionnaire (IEQ) [8]. The positive intersession experience of patients has been positively linked to therapy outcome variables in various studies [9], and it has been found to be predictive of therapy outcome using weekly retrospective measures [10-12].

More fine-grained monitoring systems such as the Synergetic Navigation System (SNS) [13] not only measure outcomes but focus on data concerning the intersession process. Ubiquitous Internet access enables such systems to draw their data from the Ecological Momentary Assessment (EMA). This approach bears detailed information that routine outcome monitoring is by design unable to detect, thereby making it possible to target interventions or to recognize the specific areas of psychotherapy that need more attention. If, for example, a patient reports a drop in perceived therapeutic relationship quality in between sessions, the therapist should focus on improving this quality in the next session. For patients, there are clear benefits as well. Their perception of problematic thoughts and feelings is trained, possibly leading to a more mindful processing of their daily experience. Problematic thoughts and feelings are validated during feedback sessions with their therapists, which may also improve the quality of the therapeutic relationship. An in-depth discussion of the advantages and possible caveats of this approach were discussed by the authors of the SNS [13].

### Dynamic Modeling of Psychopathology

Another area of psychotherapy in which monitoring systems for mental health practitioners may be important but are not yet as widespread, is diagnosis. Standard methods for clinical diagnosis of mental disorders are cross-sectional. Though diagnostic criteria for most disorders require a manifestation of symptoms over a certain period of time, ranging from weeks to several months, patients are generally assessed retrospectively at one specific point in time. Results are then typically compared with those of other patients or a specific “cutoff” score. These diagnoses are useful for classification, but they offer only limited guidance for planning interventions. Thus, individualized case formulations of psychopathology are frequently used in most psychotherapeutic orientations [14-16], offering a more complex view on individual cases and allowing practitioners to choose specific interventions.

Methodologically, this bears some problems. These methods only try to approximate temporal associations and causalities using subjective retrospective data. Recently, attempts have been made to base diagnostics on data collected in real time using EMA. For example, Fisher [17] was able to build highly individualized disorder models that were used for prescriptive treatment decisions. This approach follows four steps. First, an inventory of test items for measuring all relevant aspects of a disorder is compiled. This typically includes self-report symptom scales. Then, these items are administered using intensive repeated measurement. In practice, daily questionnaires assessing symptom severity are used. The assessment frequency can be increased to several times a day. This step results in multivariate time series data on a patient's psychopathology. To determine the synchronous associations of symptoms, factor-analysis methods are applied. The “P-technique” factor analysis is the most common approach for this step [18]. In the final step, the time-dependent relationships are assessed using multivariate time series analysis methods such as vector autoregressive (VAR) modeling. Following this diagnostic approach, Fisher thereupon presented first attempts to plan therapeutic interventions [19] based on individualized assessment.

### Objective

Until now, only commercial process and outcome monitoring applications exist. Their implementation can be costly, especially for private practices. Commercial applications cannot be extended with new functionalities by third persons. Instead, new features have to be requested from the developing company, which in turn can take considerable amount of time, depending on how much development effort is put into the requested functionalities. This makes it hard for researchers to fit the applications to their needs. Free software released under an open-source license makes the source code of the application publicly available, guaranteeing easy extension by developers who are interested in participating.

Hence, our objective was to develop an application containing the latest advancements in psychotherapy process research and dynamic assessment of psychopathology using open-source software. To accomplish this objective, we created the DynAMo (short for “dynamic assessment and modeling”) Web application. The results of this development process are presented in this paper to inform the researchers and clinicians of the results of this process.

## Methods

### The DynAMo Web Application

The presented application is based on community-driven, open-source software packages. One of its main uses is treatment planning based on the diagnostic approach that has been described previously. Treatment planning begins before the actual psychotherapy starts; hence, this function is directed at persons with mental health problems intending to seek treatment. The treatment planning function is based on an algorithm that generates patient-specific models of psychopathology from data collected in real time. These patient-specific models can be considered as an important step to a methodologically more sound and a more individualized view of psychopathology and, in practice, to treatment approaches that become more effective by being tailored to a single patient's needs. The application can also be used for monitoring processes and outcomes in psychotherapy. The DynAMo application consists of multiple modules that can run in combination but also independently. The collection of data is accomplished by a data assessment module. The treatment planning algorithm uses the collected data to generate actionable information for targeting interventions. The practitioner interface is used by clinicians to access and inspect the collected data. Researchers can use this interface to examine data collected in psychotherapy process studies.

### Data Assessment Module

**Figure 1:**
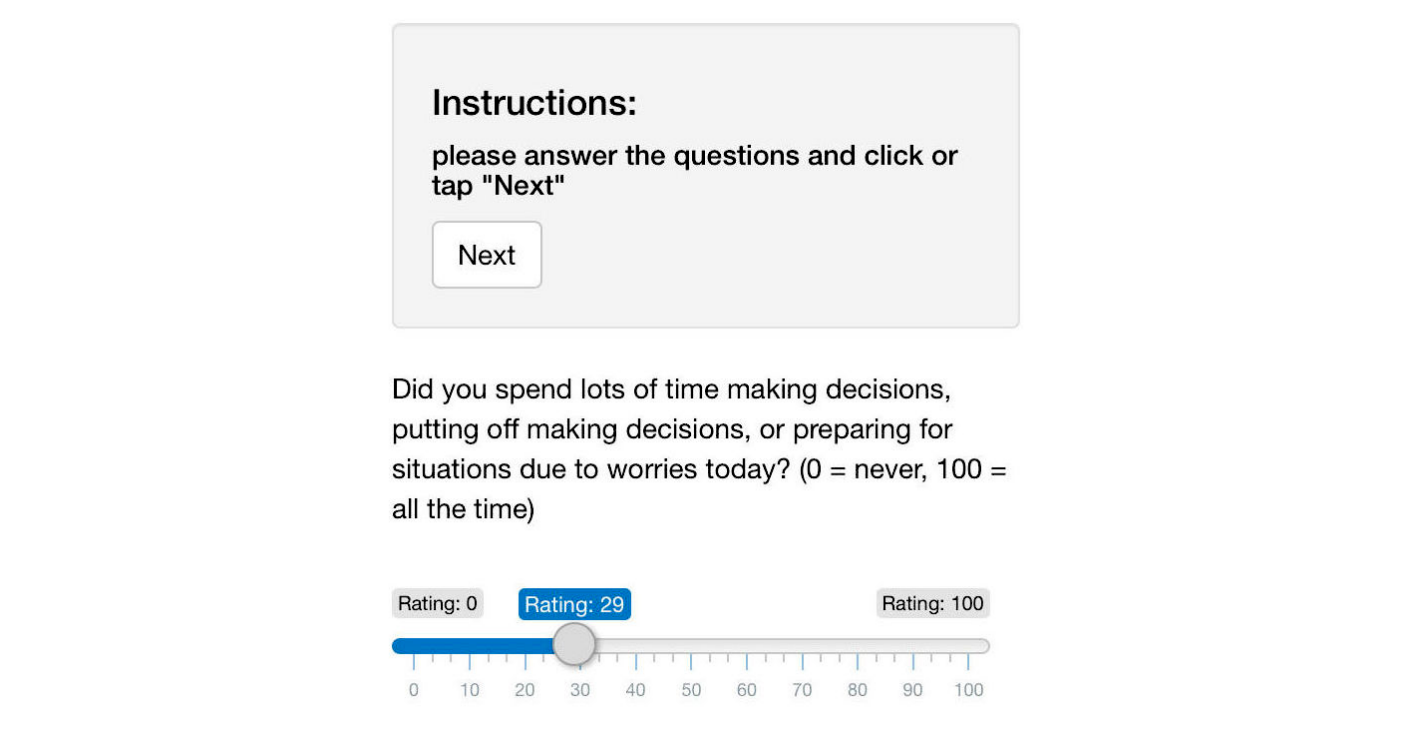
Data assessment interface on a mobile phone.

The questionnaires can be designed freely, using user interface elements from the Shiny Web framework [20]. Using the mirtCAT (computerized adaptive testing with multidimensional item response theory) package for Gnu R [21], this module of the DynAMo Web application is able to automatically send Web links to personal questionnaires at preset times. At this time, this is possible either via email or text message. Messages include a URL that leads to the questionnaire page. An example item is depicted in Figure 1. Every item has to be answered and is completed by clicking the *Next* button. After completing a questionnaire, the collected data are saved to the server. All data transmitted to and from the application are encrypted using Transport Layer Security 1.2. Data collected in the assessment are stored without any person-related data. In the configuration file, a patient code chosen by the therapist can be entered for later identification. This patient code is used for naming the database entries, so that the therapist can identify his patients when loading data. Other patient data have to be stored externally.

### General Approach to Data Collection Using DynAMo

Before a person with intention to treat can begin dynamic assessment, he/she has to meet with his/her clinician for initial diagnostic screening and an introduction to the assessment system. This is necessary to determine which symptoms should be included in the daily measures. Basically, items for the main diagnosis and any comorbidity should be included. Because of the novelty of the dynamic assessment method, there are no recommended scales. However, every self-report measure of psychopathology that is reliable and valid can be used. For many disorders, short questionnaires exist, and in the current Diagnostic and Statistical Manual of Mental Disorders, 5th edition, the American Psychiatric Association is offering a number of disorder-specific measures; for example, for various anxiety disorders, posttraumatic stress disorder, depression, acute stress symptoms, and dissociative experiences [22]. Additionally, the mirtCAT module used in this application allows to administer computer-adaptive questionnaires following item response theory (IRT). However, DynAMo currently has no option to administer psychological tests following IRT.

All items to be selected for daily assessments are then compiled to one questionnaire and included in the patient’s configuration file. If daily questionnaires are used, assessments should be scheduled for the evening. Thereby, the patient can retrospectively estimate his moods and symptoms experienced during the past day. If multiple assessments per day are planned, it is recommended to schedule them with equal temporal distances. This can be achieved by asking patients about their regular sleeping and waking times and splitting the resulting time window into equal parts. Generally, multiple assessments per day should be preferred, because higher assessment frequencies ensure a more fine-grained dataset that includes daily variation.

### Treatment Planning Algorithm

The key feature of the DynAMo application is the algorithm used for estimating the impact of treatment when a specific symptom is targeted. This information is distilled from the time series data collected from patients before starting their treatment. The algorithm provides priority scores for each assessed symptom. The algorithm analyzes data from all patients currently participating in a dynamic assessment procedure. If a model was generated successfully, notifications can be sent out to practitioners or researchers via email. These notifications can optionally include a table of symptom priority scores, so it is optional to use the practitioner interface.

Though the fundamental structure of the treatment planning algorithm was proposed by Fisher [17], it has been refined and automatized for use in DynAMo. Practitioners can use the practitioner interface to check if a model has already been identified. Every time this is checked, the algorithm will run, trying to identify a disorder model with satisfactory fit indices with the data that have been collected. The number of measurements required depends on the number of items administered and has great interindividual variation. Though in the authors’ experience 40 to 60 measurements are sufficient for some patients, this number was set to about 120 by other researchers [19]. In terms of time, exposure assessments can take from 2 to 6 weeks. The steps of the algorithm are explained in the following sections. For exemplary R code, including example data, see Multimedia Appendix 1.

#### Exploratory P-Factor Analysis

The first step in assessing the correlational structure in a patient's multivariate symptom time series is factor analysis. This is done to identify the latent dimensional structure of a patient's disorder. A maximum likelihood factor analysis is conducted with the collected time series data. Oblimin rotation is applied, because the latent dimensions of psychopathology are expected to show intercorrelation. Three models are generated simultaneously, assuming two, three, or four latent factors. Models including more than four factors are theoretically conceivable but have not yet been implemented in this application. If goodness-of-fit statistics reach satisfactory levels for any of these models, the algorithm proceeds to the next step. Goodness-of-fit measures for all factor analysis steps have been chosen according to the cutoff criteria proposed by Hu and Bentler [23]. These criteria have been determined in simulations and were found to minimize the risk of both an over- and underestimation of model fit. If more than one model reaches satisfactory goodness-of-fit measures, the model with the smallest number of factors is selected.

#### Confirmatory P-Factor Analysis

A factor loading matrix is extracted from the exploratory model. This matrix gets converted to a structural equation system that can be tested using the *lavaan* [24] package for Gnu R. Only items with factor loadings greater than .30 are included in this equation system. This step was introduced to increase statistic rigor in the algorithm. If the exploratory model is confirmed as indicated by fit measures, the algorithm proceeds to the next step.

#### Vector Autoregressive (VAR) Modeling

Vector autoregressive models are fit using the *vars* package [25] for Gnu R, following an approach first used in econometry by Lütkepohl [26]. Its use has been increasingly common in psychiatry and psychotherapy [27-30]. An application of this approach to VAR modeling to psychotherapeutic process data, including an in-depth description and basic R and Statistical Analysis System code, was provided by Ramseyer et al [30].

Factor scores for every point in assessment are generated by multiplying the raw data matrix with a weighting matrix obtained by confirmatory factor analysis (CFA). This results in a multivariate time series of factor scores. To this time series, VAR modeling is applied to determine the associations between the extracted factors. The parameters obtained in this step are:

1. Autoregressive parameters. This is a regression parameter that describes how strongly the value of a factor at one point in time (time *t−1*) is associated with the value of the same factor at a later point in time (*t*). Factors with high autoregressive parameters are relatively stable over time.
2. Cross-regressive parameters. This describes the intensity of the association of a certain factor at one point of time with another factor measured later on.
3. Linear trends. Positive or linear trends are more likely to be observed in longer time series. They indicate that the mean of the respective factor changes over time.
4. Synchronous associations. This refers to the correlation between two factors at one point in time, indicated by a correlation coefficient.
5. Whereas only auto- and cross-regressive parameters are directly relevant for the next steps, the risk to overestimate them is reduced by including the variance explained by linear trends in the model.

#### Factor Scores

The relative amount of variance (expressed in percentage) explained by each factor in the confirmatory factor model is multiplied with the relative amount of variance explained by this factor's auto- and cross-regressive parameters in the VAR model. When all factors are scored, the raw scores are standardized by dividing their scores by the score of the highest-scoring factor. This is done to determine the relevance of a patient's latent dimensions of psychopathology. If one factor explains 60% of variance and the other only 10%, then the symptoms associated with the first factor can be considered predominant. Also, if a factor strongly influences other factors, treating symptoms associated with it will more likely have beneficial effects of symptoms associated with other factors.

#### Symptom Scores

Symptom ratings are averaged and standardized by dividing each symptom's mean rating by the highest symptom mean. Means are then multiplied by the factor scores the items belong to and their loadings on this factor. Symptom scores are then standardized just like factor scores. These scores now contain a lot of information relevant for treatment planning. First, high-scoring items indicate that treating them first will result into the strongest decrease of subjective distress, because they are more likely to belong to a high-scoring factor. Second, because the item scores contain information on time-lagged associations, treating them will most likely affect other symptoms as well, as they are more likely to explain a greater amount of variance in the VAR model.

## Results

### Clinical Example for the Treatment Planning Algorithm

In order to exemplify the treatment planning algorithm, data recorded from a 30-year-old male patient currently in treatment for social anxiety disorder were used to illustrate the steps that have been described in the previous section. The patient completed a 10-item dimensional scale measuring social anxiety symptom severity [31] three times a day, at 8:00 AM, 2:00 PM, and 8:00 PM, respectively, for 5 weeks, resulting in 103 data points. The items administered are listed in Table 1.

#### Step 1: Exploratory Factor Analysis (EFA)

The algorithm is programmed to find an exploratory factor model using the least number of factors while still fulfilling the fit criteria. In this case, a two-factor model was found. The factor analysis was conducted as described in the previous section. Table 2 shows the factor loadings determined. Due to oblimin rotation, correlations between factors were allowed in the model. Factors were correlated with *r*=.56.

This factor solution suggested that the latent structure of this patient’s disorder consists of one factor mainly driven by anxiety and to some extent, by fear and avoidance, whereas items loading on the second factor described not only physical symptoms such as a racing heart and muscle tension, but also distraction and avoidance. In exploratory models, the algorithm checks the Tucker-Lewis-Index (TLI) and the root mean squared residual (RMS). Both measures were adequate for this model (TLI=.958, RMS=.056).

#### Step 2: Confirmatory Factor Analysis

Model terms for CFA are determined by the algorithm according to the EFA model structure. Only items with factor loadings greater than .30 are included in the CFA model. In this case, one item was excluded from the model due to insufficient factor loadings. The structure that is to be confirmed can be represented by the following two terms:

Factor 1 = Item 1 + Item 2 + Item 6

Factor 2 = Item 1 + Item 3 + Item 4 + Item 5 + Item 6 + Item 7 + Item 8 + Item 9

Factor loadings from the CFA model can be found in Table 3. Also, as in EFA, correlations between the factors were allowed in this model. In the CFA model, factor scores were correlated with *r*=.243.

**Table 1:** List of items in the social anxiety questionnaire administered by the example patient.

Item number	Item text, prefixed by “Since the last assessment, I have...”
1	Felt moments of sudden terror, fear, or fright in social situations.
2	Felt anxious, worried, or nervous about social situations.
3	Had thoughts of being rejected, humiliated, embarrassed, ridiculed or offending others.
4	Felt a racing heart, sweaty, trouble breathing, faint, or shaky in social situations.
5	Felt tense muscles, felt on edge or restless, or had trouble relaxing in social situations.
6	Avoided, or did not approach or enter, social situations.
7	Left social situations early or participated only minimally (eg, said little or avoided eye contact)
8	Spent a lot of time preparing what to say or how to act in social situations.
9	Distracted myself to avoid thinking about social situations.
10	Needed help to cope with social situations (eg, with alcohol, medications, or superstitious objects).

**Table 2:** Factor loadings in the exploratory model. Loadings smaller than .10 were omitted.

Item number	Factor 1	Factor 2
1	.31	.40
2	.97	-
3	-	.58
4	-	.57
5	-	.65
6	.34	.40
7	-	.43
8	-	.49
9	-	.56
10	-	-

**Table 3:** Factor loadings resulting from confirmatory factor analysis.

Item number	Factor 1	Factor 2
1	.569	.129
2	.810	-
3	-	.624
4	-	.519
5	-	.657
6	.626	.094
7	-	.368
8	-	.670
9	-	.437

The TLI and the standardized root mean squared residual (SRMR) is checked for confirmatory models. Both measures were adequate for this model (TLI=.959, SRMR=.05). Thus, the model found with EFA was confirmed with increased statistical rigor, and the algorithm could proceed.

#### Step 3: Vector Autoregressive (VAR) Model

**Figure 2:**
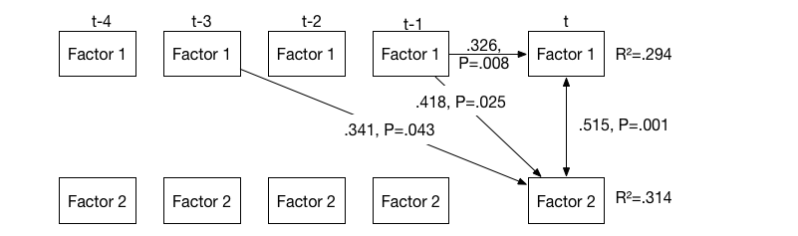
Vector autoregressive model, including auto- and cross-regressive parameters (one-headed arrows) and a synchronous association (double-headed arrows). Only statistically significant parameters are shown. Explained variance is indicated by R-squared values.

For this step, factor scores are extracted from the time series by multiplying the raw scores with the factor loading matrix. In this example, this resulted in a time series of 103 points of measurement for each factor. From these time series, a VAR model is computed to determine time-lagged associations between factor scores. The number of lags is determined by comparing the Akaike information criterion (AIC) for models with one to five lags, choosing the number that leads to the lowest AIC value. In this case, four lags were chosen. Regression models for both factors explained a statistically significant amount of variance (R^2^=.294, *F*_9,89_=5.527, *P*=5.011^-6^) for the first factor and (R^2^=.314, *F*_9,89_=5.979, *P*=1.63^-6^) for the second factor. Figure 2 shows a graphical depiction of the model. The VAR model shows that both factors show time-lagged associations with factor 1. From this model, it can be concluded that symptoms associated with the first factor have significant influence on the severity of symptoms associated with the second factor. In this example, an increased level of anxiety, worry, or nervosity about social situations as well as sudden fright and terror in social situations and avoidance were associated with the first factor. If these symptoms increase, symptoms associated with the second factor (an increased level of somatic symptoms such as muscle tension, a racing heart and sweat, or more cognitive symptoms such as preparation for social situations or thoughts about being ridiculed or humiliated) are more likely to increase. Because there are significant vector regressive parameters from the three lags and the patient completed a symptoms questionnaire three times a day, this influence was measurable from measurements up to 24 hours ago. The first factor also has a significant auto-regressive component, meaning that symptoms associated with the first factor are more stable over time.

#### Step 4: Factor Scores

After the time-lagged associations between factor values have been determined, the algorithm proceeds to determine the relevance of the two factors for the patient’s psychopathology. First, the squared loadings of each item in the CFA model are divided by the number of factors and summed up. The result is the explained amount of variance for the respective factor. This value represents the amount of variance a factor explains at one point in time. The same is done with the auto- and cross-regressive parameters in the VAR model, leading to a value indicating the amount of explained variance between several points in time. In this case, the first factor explained 34.27% of within-time variance and 22.96% between-time variance. The second factor explained 47.27% within-time variance and 6.1% of between-time variance. Both types of variance are then multiplied and normalized by dividing them by the largest resulting value. The two factor scores resulting from this step were 1 and .367.

#### Step 5: Symptom Scores

In the last step, means for each item from the social anxiety scale are calculated and normalized by dividing them by the largest item mean. Then, each item’s factor loading is multiplied with the factor score. If an item loads on more than one factor, this is done for every factor the item loads upon. The resulting value is multiplied with the item’s normalized mean. Now, every item has a symptom score containing information about their average severity, their contribution to each factor, and the relevance of the factor the item contributes to. Finally, these item scores are normalized by dividing them by the highest item score and multiplying them by 100. The result is a priority rating for each item. Table 4 shows the items and their symptom scores.

**Table 4:** Symptom scores resulting from the treatment planning algorithm, sorted by maximum to minimum priority.

Item text	Normalized symptom score
Felt anxious, worried, or nervous about social situations.	100
Spent a lot of time preparing what to say or how to act in social situations.	95.43
Felt a racing heart, sweaty, trouble breathing, faint, or shaky in social situations.	44.13
Avoided, or did not approach or enter, social situations.	41.58
Felt moments of sudden terror, fear, or fright in social situations.	36.78
Had thoughts of being rejected, humiliated, embarrassed, ridiculed or offending others.	29.86
Felt tense muscles, felt on edge or restless, or had trouble relaxing in social situations.	28.61
Left social situations early or participated only minimally (eg, said little, avoided eye contact)	23.80
Distracted myself to avoid thinking about social situations.	21.21

The result obtained from applying the treatment planning algorithm to the patient’s data suggests that the treatment targets the first two symptoms listed in table 4: anxiety, worry and nervous feelings about social situations and the excess amount of time that the patient spends preparing for social situations. This could be done by combining a relaxation exercise with imaginal exposure techniques. Self-control desensitization [32] is a well-validated approach that would cover this combination. Note that, after treatment has proceeded for some time and the recording of symptom scores is continued, a new model might be found by the algorithm, prioritizing different symptoms and thus, suggesting a change in the treatment plan.

### Practitioner Interface

The practitioner interface was developed using the Shiny Web framework. This allows combining the powerful statistical computing and plotting capabilities of the R programming language with a well-developed Web framework. The application consists of two modules that can run independently: the practitioner interface designed for convenient data interpretation and the assessment interface that delivers questionnaires to patients. Practitioners can review patient data using a convenient and intuitive Web interface.

### Treatment Planning Information

The summary of item scores produced by the treatment planning algorithm can be inspected in the “Treatment Planning” tab. A table (similar to Table 1) with all symptoms that have been assessed is shown, including a standardized priority score that results from the treatment planning algorithm.

### Time-Series Inspection

**Figure 3:**
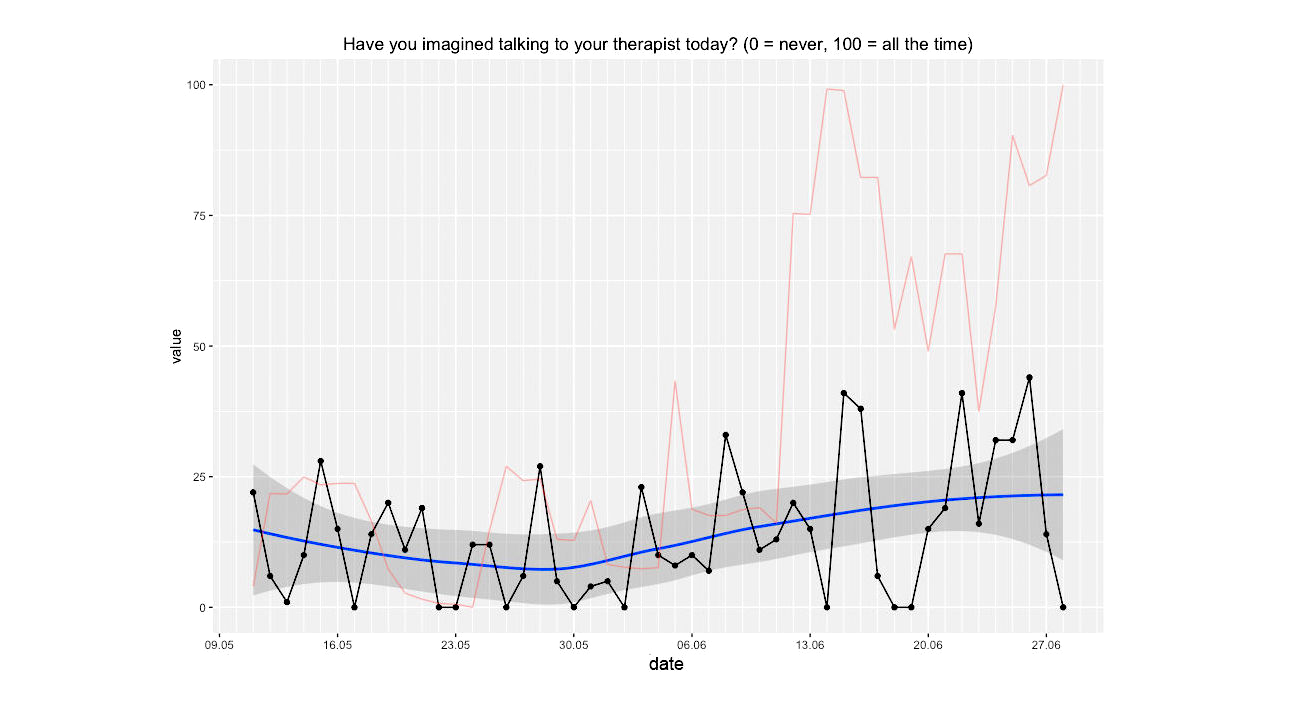
Time series plot showing answers to an intersession process item. A line smoothed by local regression scatterplot smoothing (LOESS) is added for easy interpretation of long-term change, including the 95% CI. The red line represents the measure of dynamic complexity. The x-axis represents the date of measurement (day and month).

Individual time series for every item assessed by DynAMo can be inspected via the practitioner interface. As depicted in Figure 2, different auxiliary plots are available:

- A local regression curve graph with 95% CI for easy interpretation of shifts in means.
- A plot of the *dynamic complexity* of the time series. Dynamic complexity is composed of the intensity of fluctuation and the degree of distribution of values in a moving window of a time series [33]. Typically, this window has a width of 5 to 7 data points. Flat curves with a few different values result in low dynamic complexity, whereas curves that oscillate strongly and include a variety of different values result in high complexity. Local peaks of dynamic complexity indicate critical instabilities of a system, which are likely to be accompanied by sudden changes in the system’s components, so-called *phase transitions* [34]. Applied to psychotherapy, critical instabilities often precede symptom changes if “boundary conditions” such as a positive therapeutic relationship are fulfilled [35]. Hence, this plot offers additional information that can be interpreted alongside the raw data curve. In this application, the dynamic complexity is rescaled to fit the theoretical maximum and minimum of the raw plot.
- As illustrated in Figure 3, the application is able to generate the so-called *recurrence plots* [36] to further ease the interpretation of time series for the trained user. Recurrence plots visualize the Euclidean distance between points in a time series so that the recurring patterns in time series data become more obvious. This is achieved by plotting the Euclidean distance in time x time diagrams. Similar to dynamic complexity, recurrence plots are used to identify phases of critical instability. With recurrence plots, rare or far-from-normal states in a time series can be identified, which are indicative of occurring phase transitions. Recurrence plots are optional and not plotted by default in this application. However, their use in the monitoring of psychotherapy processes is quite common in comparable applications [13]. 

Summarized, when using DynAMo, practitioners can extract a large amount of information on their patients by analyzing their time series. No complicated mathematical operations have to be carried out by the practitioners themselves because all of the information contained in a time series is represented graphically.

### Clinical Example

This example illustrates process monitoring of a 22-year-old female patient suffering from bulimia nervosa, currently undergoing Rogerian person-centered psychotherapy.

The psychotherapy process was measured daily, using a short form of the German version of the IEQ [8]. This questionnaire measures therapy-related cognitions, emotions, and behaviors. The *Reflecting treatment* scale measures thoughts about the patient's behavior toward psychotherapy. The scale named *Relationship fantasies* indicates the frequency of thoughts involving the therapist. The “Problem solving” scale contains items remembering therapy contents and applying them in the patients’ daily routine. Also, *Therapy-related emotions* are measured, reflecting positive and negative emotions toward the current therapy.

Weekly therapy outcome was measured on Sundays, using a 27-item short form of the Symptom Checklist-90 (SCL-90) in German [37,38]. Weekly pre-post change scores were calculated.

**Figure 4:**
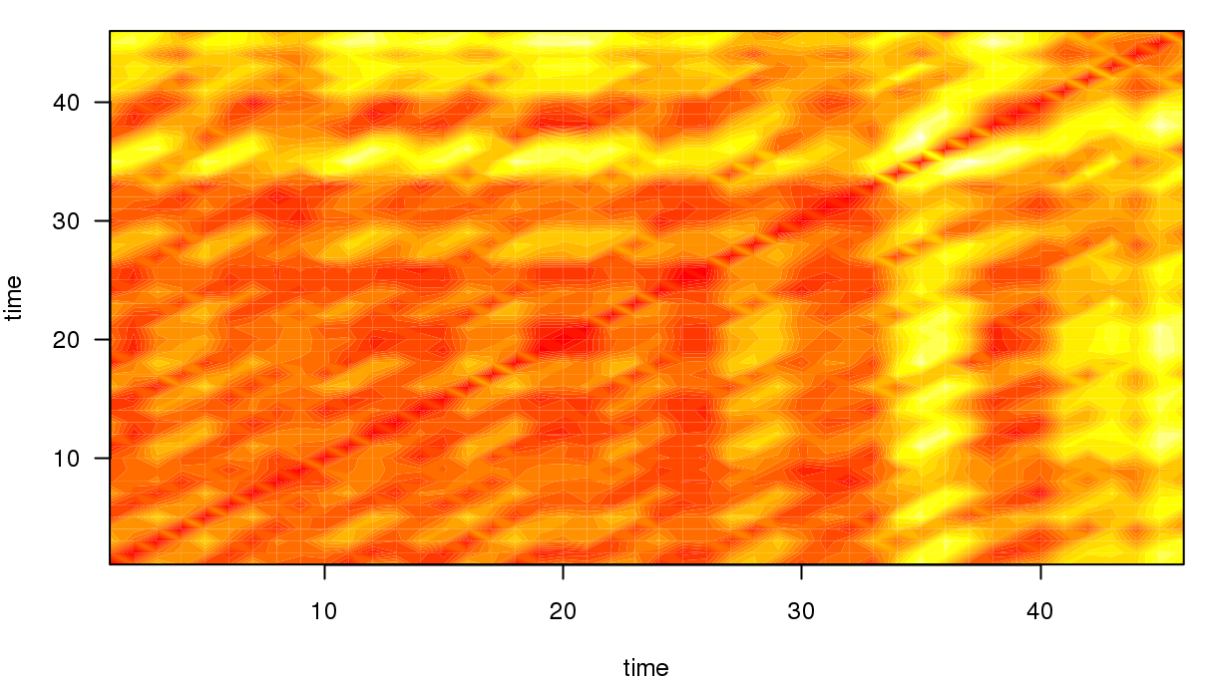
Recurrence plot for the time series illustrated in Figure 2. Darker, red-colored areas of the plot indicate low Euclidean distance between the respective points of the time series whereas brighter, more yellow areas indicate greater Euclidean distance. Greater distance also implies that the time series currently describes a period that has not occurred before. Note that the periods of increased dynamic complexity in the time series depicted in Figure 2 are reflected in the recurrence plot. Both “time” axes refer to points of measurement in the time series.

A depicted in Figure 4, the patient experienced two local peaks of dynamic complexity in her “Problem Solving” scale. The first peak, measured in the first week of treatment, was followed by a symptom reduction of 15%. One week later, another 5% of symptom reduction was observed. The second major peak was measured in the third and fourth week of treatment. Initial weekly treatment outcome was a 2% increase in symptom severity; this was followed by an 11% decrease in the following week. Time-lagged change in outcome measures is a common pattern observed when associating them with periods of complexity [33]. In the fifth and sixth week of treatment, complexity scores dropped and no peaks occurred. Also, a 34% increase in symptoms was observed, followed by another slight increase.

The observed peaks of complexity can be interpreted as ongoing processes of change, which can be useful in anticipating changes. Practitioners observing these peaks would be well-advised to ensure that the patient experiences stability and a positive therapeutic relationship in her therapy sessions.

However, the increase in symptoms in the fifth week cannot be explained by inspecting only one curve. Additional information can be drawn from other curves. As illustrated in Figure 6, the outcome measure on June 4 was associated with a significant decrease in positive treatment-related emotions (eg, *relief, hope, secure*) and an increase in negative emotions (eg, *anxious, frustration, sadness, hurt*). This information can be used for clarifying the negative outcome reported by the patient.

This relatively simple example shows how therapy process data can inform clinicians about their patients’ thoughts and feelings toward their therapy. Information on periods of change the patient is going through as well as negative evaluations of therapy progress would not be accessible to the therapist, possibly leading to missed opportunities to course correction of a therapy that went “off track.”

**Figure 5:**
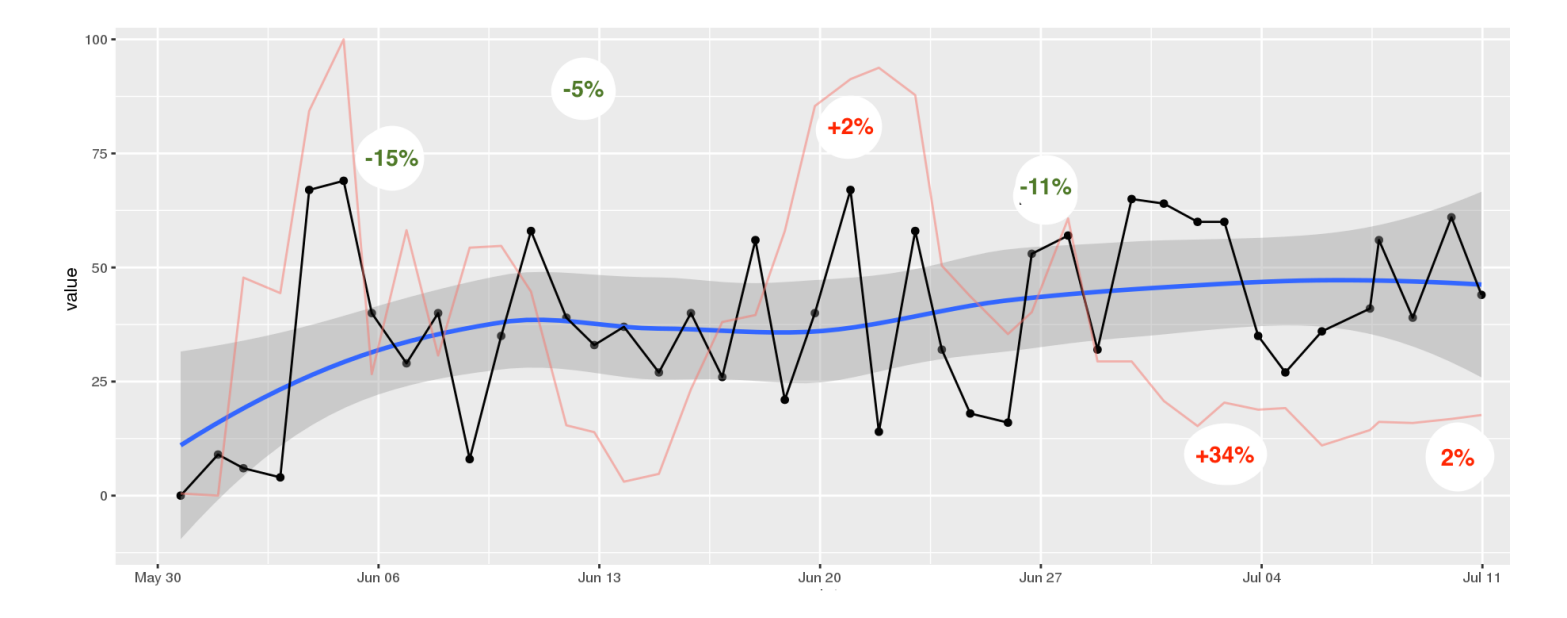
Time series plot showing mean scores of the “Problem Solving” scale of the short intersession questionnaire. A line smoothed by local regression scatterplot smoothing (LOESS), including the 95% CI is added for easy interpretation of long-term change. The red line represents the measure of dynamic complexity that was rescaled from 0 (minimum complexity) to 100 (maximum complexity). Percentage values indicate weekly treatment outcome in percent symptom change.

**Figure 6:**
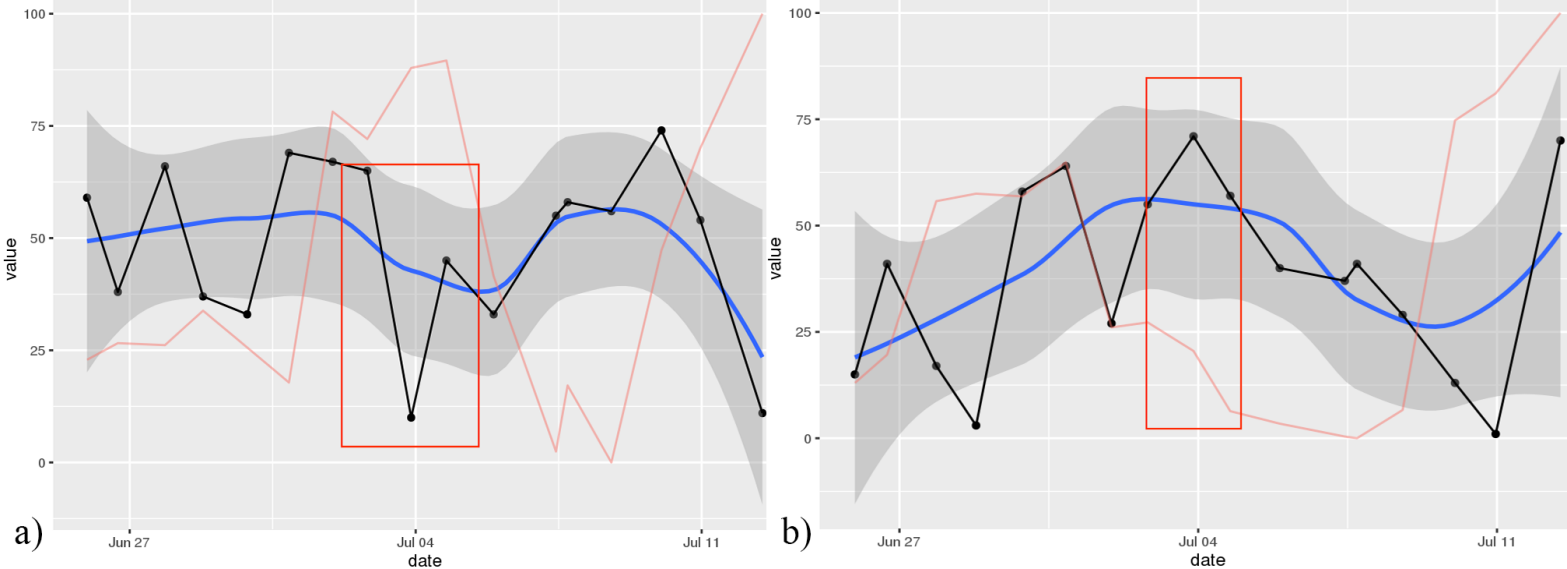
Time series plot showing the (a) the “positive treatment-related emotion” and (b) the “negative treatment-related emotion” scores. The red box marks a significant drop in positive treatment-related emotions, as well as an increase in negative ones.

## Discussion

### Limitations and Challenges

In his recent discussion of challenges in implementing psychotherapy monitoring systems, Boswell [39] identified four core obstacles for mental health providers: financial burden, time burden, different needs of different stakeholders, and turnover. We consider the software presented in this paper an attempt to tackle these obstacles. First of all, because it was developed as an open-source project, the DynAMo software package can be provided without licensing fees.

Practitioners seeking to implement process- and outcome monitoring applications face the challenge of structural changes in their day-to-day routine. The application also tries to keep time and energy expenditure as low as possible for clinicians. We developed the software keeping in mind that it will be used by mental health practitioners, paying great attention to a user-friendly design. A first study of usability of the practitioner and treatment planning interfaces in clinical practice is currently starting. We acknowledge that there is no process monitoring application that is suitable for everyone.

Despite the easy-to-use Web interface, therapists need to be trained in the use of the DynAMo software so that they can extract useful information from patient data. Training should include tutorials and exercises on time series interpretation, including an understanding of the dynamic systems approach to change, which should include interpretation of dynamic complexity and the recognition phase transitions. This way, practitioners can easily grasp information from their patient’s trajectories and learn how to recognize periods of change. Training should also include several clinical examples so that practitioners understand how to learn from their patients’ data. Another crucial skill is offering feedback to patients so that they learn to view process and outcome monitoring as a part of their treatment. A training program including these different elements is currently developed in our research group.

Another challenge lies in data collection. Psychotherapy patients could find it difficult to answer daily questionnaires, especially if suffering from more severe disorders. Compliance rates for process monitoring are reduced by delayed starting of questionnaires, early termination, or not filling out a questionnaire at all. A feasibility study by Schiepek [40] showed an average compliance rate of 78.3% and average missing data rates of about 13% when using a 42-item daily questionnaire in an inpatient setting. Similar values were found when assessing adherence to daily mobile phone-based assessment of a short depression scale [41]. Although there is no published data for outpatients yet, modern Internet-enabled devices such as mobile phones, tablet computers, or laptops greatly facilitate data collection. Internal pre-tests of the DynAMo application showed similar compliance rates (80%) and missing data rates (10%) for outpatients. These rates can be considered satisfactory as they do not reduce data quality to a large extent. On a more general level, a study by Torous [42] could show that about 76% (n=100) of persons in the age group of 18 to 60 years are interested in monitoring their mental health with mobile applications.

Therapists’ acceptance of technology such as process monitoring applications can depend on several factors. Confirming the issues brought up by Boswell [39], research on technology acceptance [43,44] could show that expected performance of the software is a strong predictor of the intention to use it. Thus, clinical practitioners could feel that the proposed software performs not well enough for routine use. In future usability studies, we will carefully review the data provided by therapists.

Also, the effort to use the software predicts this intention, stressing the importance of usability and training sessions. Practitioners could however see training sessions as a burden, consuming excess time; hence, it will be necessary to design the software so that training sessions can be reduced to a minimum.

Influence by coworkers has been found to increase usage, especially for users with limited experience. It can be concluded that training besides training sessions and regular meetings of users could foster the adoption of this technology. In these meetings, users can also discuss experiences and possible issues using this software.

The intent of this paper was to present a series of tools for psychotherapy process research to the community. Thus, empirical data was only used for illustrating the software’s features, offering only a limited view on the possibilities of this software package. It will be the focus of future empirical studies to obtain larger data sets, including usability data provided by clinical practitioners.

### Outlook

It was the goal of this project to provide the psychotherapy research community with a set of tools to study the processes and mechanisms of change in psychotherapy with the high temporal resolution needed to get ecologically valid results and without depending on costly alternatives. We presented a newly developed software for psychotherapy process monitoring and treatment planning in mental health settings. Whereas parts of the software are still under development, the base set of features is complete, and it can now be considered ready for application in empirical research and clinical practice. The DynAMo software should be viewed as an evolving toolset and the full source code is to be released under an open-source software license at a future date via a public project hosting platform such as GitHub, inviting other developers and researchers to participate.

The treatment planning algorithm makes it possible to tailor therapeutic interventions to individual patients, appreciating the great complexity of psychopathology and psychotherapy. With the DynAMo application, the therapy process can be monitored, so that important periods of change are transparent to the therapist. This includes identifying periods of change in ongoing therapies, both from a linear point of view using smoothed graphs and from a dynamic systems point of view, using dynamic complexity plots. The functions of this application cover the period before starting psychotherapy, psychotherapy itself, and they can also be used as a means of sustaining change and preventing relapse by monitoring symptoms after completion for a certain period of time. Thus, the presented application is not only a research tool but also a tool for enhancing psychotherapy with new technologies.

Next steps in the development process of the DynAMo application include an administrator’s interface that features easy creation of patient configurations and editing of assessment items using a graphical user interface. Another feature is the possibility of adding short, free-form text items that can be viewed as annotations in time-series graphs. This way, patients can report on meaningful events in a more detailed way. Also, “Traffic-light”-style notifications for certain critical items will be introduced in the future. These notifications can warn therapists of possible treatment drop-out, self-harming behavior, or other critical incidents and also inform them about beneficial developments such as increases in working alliance quality or successes while applying behaviors learned in therapy. Both therapists’ and patients’ experience will be recorded and examined, so that the application can adapt to their needs.

## Appendix

Multimedia Appendix 1 This example script will demonstrate the treatment planning algorithm in the paper "A Modular Platform for Monitoring Process, Outcome and Algorithm-Based Treatment Planning in Psychotherapy". All steps are illustrated using a simulated data set with items on social anxiety included in the ZIP file. An additional text file with item texts is included as well.

## Abbreviations

AIC: Akaike information criterion
CFA: confirmatory factor analysis
DynAMo: dynamic assessment and modeling
EFA: exploratory factor analysis
EMA: Ecological Momentary Assessment
IEQ: Intersession Experience Questionnaire
IRT: item response theory
mirtCAT: computerized adaptive testing with multidimensional item response theory
OQ: Outcome Questionnaire
PCOMS: Partners for Change Outcome Management System
RMS: root mean squared residuals
SNS: Synergetic Navigation System
SRMR: standardized root mean squared residual
TLI: Tucker-Lewis-Index
VAR: vector autoregressive
